# Probiotics for treating acute infectious diarrhoea

**DOI:** 10.1590/S1516-31802011000300012

**Published:** 2011-05-05

## Abstract

**BACKGROUND:**

Probiotics may offer a safe intervention in acute infectious diarrhoea to reduce the duration and severity of the illness.

**OBJECTIVES:**

To assess the effects of probiotics in proven or presumed acute infectious diarrhoea.

**SEARCH STRATEGY:**

We searched the Cochrane Infectious Diseases Group's trials register (July 2010), the Cochrane Controlled Trials Register (*The Cochrane Library* Issue 2, 2010), MEDLINE (1966 to July 2010), EMBASE (1988 to July 2010), and reference lists from studies and reviews. We also contacted organizations and individuals working in the field, and pharmaceutical companies manufacturing probiotic agents.

**SELECTION CRITERIA:**

Randomized and quasi-randomized controlled trials comparing a specified probiotic agent with a placebo or no probiotic in people with acute diarrhoea that is proven or presumed to be caused by an infectious agent.

**DATA COLLECTION AND ANALYSIS:**

Two reviewers independently assessed the methodological quality of the trial and extracted data. Primary outcomes were the mean duration of diarrhoea, stool frequency on day 2 after intervention and ongoing diarrhoea on day 4. A random-effects model was used.

**MAIN RESULTS:**

Sixty-three studies met the inclusion criteria with a total of 8014 participants. Of these, 56 trials recruited infants and young children. The trials varied in the definition used for acute diarrhoea and the end of the diarrhoeal illness, as well as in the risk of bias. The trials were undertaken in a wide range of different settings and also varied greatly in organisms tested, dosage, and participants’ characteristics. No adverse events were attributed to the probiotic intervention. Probiotics reduced the duration of diarrhoea, although the size of the effect varied considerably between studies. The average of the effect was significant for mean duration of diarrhoea (mean difference 24.76 hours; 95% confidence interval 15.9 to 33.6 hours; n = 4555, trials = 35) diarrhoea lasting ≥ 4 days (risk ratio 0.41; 0.32 to 0.53; n = 2853, trials = 29) and stool frequency on day 2 (mean difference 0.80; 0.45 to 1.14; n = 2751, trials = 20). The differences in effect size between studies was not explained by study quality, probiotic strain, the number of different strains, the viability of the organisms, dosage of organisms, the causes of diarrhoea, or the severity of the diarrhoea, or whether the studies were done in developed or developing countries.

**AUTHORS’ CONCLUSIONS:**

Used alongside rehydration therapy, probiotics appear to be safe and have clear beneficial effects in shortening the duration and reducing stool frequency in acute infectious diarrhoea. However, more research is needed to guide the use of particular probiotic regimens in specific patient groups.

**Flavio Steinwurz. Scientific Coordinator of the Department of Gastroenterology, Associação Paulista de Medicina (APM).**

This is the abstract of a Cochrane Review published in the Cochrane Database of Systematic Reviews (CDSR) 2010, Issue 11, DOI: 10.1002/14651858.CD003048.pub3. (www.thecochranelibrary.com). For full citation and authors details see reference 1

**For Latin America and the Caribbean, the full text is freely available from:** http://cochrane.bvsalud.org/cochrane/show.php?db=reviews&mfn=1537&id=CD003048&lang=pt&dblang=&lib=COC&print=yes

**For other regions, the abstract is available from:** http://onlinelibrary.wiley.com/o/cochrane/clsysrev/articles/CD003048/frame.html

**This section was edited under the responsibility of the Brazilian Cochrane Center**

## COMMENTS

Probiotics are nonpathogenic live microorganisms that provide beneficial effects for the host's health. Over recent years, they have been used to treat infectious diarrhea, since some studies have shown that their use may decrease the severity and duration of this disease. Probiotics help to improve the balance of the intestinal microflora, but their mechanism of therapeutic action remains unclear. They possibly act through suppressing the growth and invasive power of pathogenic bacteria, or though improving the protective capacity of the intestinal wall barrier, or even through an immunological mechanism. The literature shows that they have beneficial effects in some cases of diarrhea caused by rotavirus, especially in children.

The authors’ review^1^ is a very interesting paper, since it covered 63 studies with over 8,000 patients, with an excellent scientific analysis.

The vast majority of cases of infectious diarrhea are self-limited and do not require any treatment at all. Since administration of antiemetics and antidiarrheal agents is not recommended in these cases, particularly for children with acute infectious diarrhea, probiotics may be a good option in mild cases that do not need a more aggressive intervention.

It is very important to point out that not all nonpathogenic bacteria can be considered to be probiotics. There are rules and characteristics for this. Just a few strains have shown some benefit for treating diarrhea, in clinical tests, and therefore only these strains can reasonably be recommended. Further studies are needed to ensure the best indication and use of probiotics.
